# Feasibility of developing reliable gene expression modules from FFPE derived RNA profiled on Affymetrix arrays

**DOI:** 10.1371/journal.pone.0203346

**Published:** 2018-08-31

**Authors:** Vinu Jose, Debora Fumagalli, Françoise Rothé, Samira Majjaj, Sherene Loi, Stefan Michiels, Christos Sotiriou

**Affiliations:** 1 Breast Cancer Translational Research Laboratory, Institut Jules Bordet, Université Libre de Bruxelles, Brussels, Belgium; 2 Breast International Group, Institut Jules Bordet, Université Libre de Bruxelles, Brussels, Belgium; 3 Division of Research and Cancer Medicine, Peter MacCallum Cancer Centre, University of Melbourne, Melbourne, Australia; 4 Service de Biostatistique et D’Epidémiologie, Gustave Roussy, CESP, U1018, Université Paris-Sud, Faculté de Médcine, Université Paris-Saclay, Villejuif, France; 5 Department of Medicine, Medical Oncology Clinic, Institut Jules Bordet, Université Libre de Bruxelles, Brussels, Belgium; Universita degli Studi di Torino, ITALY

## Abstract

The reliability of differential gene expression analysis on formalin-fixed, paraffin-embedded (FFPE) expression profiles generated using Affymetrix arrays is questionable, due to the high range of percent-present values reported in studies which profiled FFPE samples using this technology. Moreover, the validity of gene-modules derived from external datasets in FFPE microarray expression profiles is unknown. By generating matched gene expression profiles using RNAs derived from fresh-frozen (FF) and FFPE preserved breast tumors with Affymetrix arrays and FF/FFPE RNA specific amplification-and-labeling kits, the reliability of differential expression analysis and the validity of gene modules derived from external datasets were investigated. Specifically, the reliability of differential expression analysis was investigated by developing de-novo ER/HER2 pathway gene-modules from the matched datasets and validating them on external FF/FFPE gene expression datasets using ROC analysis. Spearman's rank correlation coefficient of module scores between matched FFPE/frozen datasets was used to measure the reliability of gene-modules derived from external datasets in FFPE expression profiles. Independent of the array/amplification-kit/sample preservation method used, de-novo ER/HER2 gene-modules derived from all matched datasets showed similar prediction performance in the independent validation (AUC range in FFPE dataset; ER: 0.93–0.95, HER2: 0.85–0.91), except for the de-novo ER/HER2 gene-module derived from the FFPE dataset using the 3'IVT kit (AUC range in FFPE dataset; ER: 0.79–0.81, HER2: 0.78). Among the external gene modules considered, roughly ~50% gene modules showed high concordance between expression profiles derived from matching FF and FFPE RNA. The remaining discordant gene modules between FF and FFPE expression profiles showed high concordance within matching FF datasets and within matching FFPE datasets independently, implying that microarrays still require improved amplification-and-sample-preparation protocols for deriving 100% concordant expression profiles from matching FF and FFPE RNA.

## Introduction

Using Affymetrix arrays with formalin-fixed, paraffin-embedded (FFPE) specific sample preparation protocols, many studies reported the feasibility of generating reliable gene expression profiles from FFPE samples [1–7]. Though Affymetrix recommends similar percent-present values between samples for reliable analysis [8], most of these studies reported a high range of percent-present values [1–6], reflecting the varying proportion of reliable genes in FFPE expression profiles. This high range of percent-present values raises concerns over the reliability of differential expression analysis performed on FFPE expression profiles and the gene signatures or gene-modules developed from them. Furthermore, these studies did not analyze the reliability of gene-modules derived from external datasets (external gene-modules) in FFPE derived expression profiles.

By generating matched gene expression profiles (matched datasets) using RNAs derived from fresh-frozen (FF) and FFPE preserved breast tumors with Affymetrix arrays and FF/FFPE RNA specific amplification-and-labeling kits, the reliability of differential expression analysis and the validity of gene modules derived from external datasets were investigated. A probe set filtering strategy suitable for FFPE derived expression profiles has been used to account for the varying proportion of reliable genes present in FFPE material. The reliability of differential gene expression analysis was evaluated by recreating already established Estrogen Receptor (*ER/ESR1*) and Human Epidermal Growth Factor Receptor 2 (*HER2/ERBB2*) pathway gene-modules [9] and validating them in external datasets [10,11]. To investigate the reliability of external gene modules on FFPE expression profiles three types of comparisons (ref-1/ref-2/ref-3) were performed depending on the dataset which is used as a reference for comparisons. Specifically, the module scores computed from the reference dataset were correlated modules scores from relevant matched datasets. Using a similar approach (ref-1/ref-2/ref-3) gene and sample level correlations between matched datasets were also investigated.

## Materials and methods

### Array selection

Since in the oncology research field many collaborative trial groups have extensive collections of FPPE samples of patients included in randomized clinical trials, we specifically looked for an array which is economical for profiling a large number of FFPE tumors using an automated preprocessing system. Affymetrix HG-U219 Perfect-Match(PM) only array plate in its 96 well format seemed to be well suited for our purpose. Hence we included this array in this study for profiling both frozen and FFPE RNAs. An improved gene sensitivity (12%, ~600 genes) of the Human Exon 1.0 ST PM only array over the HG-U133plus2 Perfect-Match MisMatch (PM-MM) array has been reported for FFPE RNA with the FFPE specific kit, Nugen's Ovatio^n^® FFPE WTA System and Encore^TM^ Biotin Module [3]. However, due to the economic impact in using exon arrays for profiling a large number of archived FFPE specimens, we considered its improved gene sensitivity as marginal, and we excluded these arrays from this study. For RNAs extracted from frozen samples, Affymetrix's HG-U133plus2 PM-MM array with the 3'IVT kit is considered as the gold standard in gene expression profiling and hence was included in this study.

### Experimental design and samples details

Five sample-matched gene expression datasets were generated using RNAs derived from FF/FFPE preserved breast tumors (n = 8) with either HG-U133plus2 arrays with 3’IVT kit or HG-U219 arrays with FFPE RNA specific kits as listed below (S1 Table and S1 Fig): 1) Two datasets from matched FFPE and frozen samples profiled on HG-U133plus2 arrays using the Affymetrix's 3'IVT kit (u133p2.3ivt.ffpe/ff), 2) Two datasets from matched FFPE and frozen samples profiled on HG-U219 arrays using the Nugen's Ovation^®^ FFPE WTA System and Encore^TM^ Biotin Module (u219.ovation.ffpe/ff), and 3) a single dataset from the same FFPE samples profiled on HG-U219 arrays using the Affymetrix's SensationPlus^TM^ FFPE Amplification and 3'IVT Labeling Kit (u219.sensation.ffpe). To investigate the gene and sample level correlations between matched datasets and the reliability of external gene modules on FFPE expression profiles three types of comparisons (ref-1/ref-2/ref-3) were performed depending on the dataset which is used as a reference for comparisons (S2 Fig). In ref-1 comparison, per gene/sample expression profiles and module scores of external gene modules from all matched datasets were correlated to the gold standard expression dataset generated from frozen samples with HG-U133plus arrays and 3’IVT kit (u133p2.3ivt.ff). In ref-2 comparison, the HG-U219 dataset derived from FF RNA with ovation kit (u219.ovation.ff) was considered as reference and similar measurements as in ref-1 comparison were correlated to matched HG-U219 FFPE datasets with ovation/sensation kit. Also, in ref-3 comparison, similar measurements as in ref-1 comparisons were correlated between the two HG-U219 FFPE datasets with ovation and sensation kits.

The eight BC tumors include two tumors from each Luminal-A (ER+, HER2-), Luminal-B (ER+, HER2+), HER2-amplified (ER-, HER2+) and Triple-Negative (ER-, HER2-) subtypes; detailed sample demography is given in S2 Table. Both FFPE and frozen tumor specimens from surgically removed breast carcinoma were obtained using routine diagnostic procedures; the study was approved by the ethical committee of Institut Jules Bordet.

### RNA Extraction, hybridization and normalization

RNA was extracted from frozen samples using the TRIzol® Reagent [Thermo Fisher Scientific, Waltham, MA, USA] and from FFPE samples using the miRNeasy FFPE Kit [QIAGEN, Hilden, Germany], following the manufacturer’s instructions. RNA from matched samples was processed and hybridized on a particular combination of amplification-and-labeling kit/array according to the manufacturer's protocols (see the “Experimental design and samples details” section). Each expression dataset thus obtained was normalized independently using RMA background correction [12], quantile normalization [13] and median polish summarization [14] using its R implementation [15] available from Bioconductor [16] package affy [17]. The raw and processed expression data are available on GEO [18] under the accession id GSE93338.

### Detection calling

Present-absent calls from the HG-U133plus2 array were generated using an R implementation of the detection calling algorithm available as part of the MAS 5.0 software package [17,19], at 5% significance level. Since the detection calling algorithm from MAS 5.0 package requires matching Perfect Match—Mismatch (PM-MM) probe pairs to generate detection calls, it cannot be applied to PM only HG-U219 array. Hence a Wilcoxon rank-sum test [20] based algorithm has been proposed to generate detection calls in the HG-U219 arrays by exploiting the 23 anti-genomic background probe sets present in this array. Details of the proposed detection calling algorithm are given in the S1 File.

### Probe set filtering

We considered only non-specific filtering strategies (all-absent, variance-based [21] and PVAC [22]), as Bourgon et al. [23] showed that non-specific probe set filtering is superior to the class label based probe set filtering. Due to the high range of percent-present values in FFPE datasets, all-absent and variance based filtering may not be appropriate hence excluded from the analysis. On the contrary, the PVAC filtering method seems to be well suited for FFPE datasets, as it uses a measure of per probe-set probe-level agreement to filter probe sets [22]. The PVAC algorithm requires a set of probesets which are not expressed in the entire dataset to derive a cutoff to separate the probesets in the array as reliable and non-reliable. The original algorithm implements a strategy which depends on detection calling and considers the probesets which are absent in all samples as negative probesets. However the PM only HG-U219 array has an explicit set of 23 anti-genomic probesets used to measure background expression, and these probesets can be used as negative probesets for PVAC probeset filtering. This approach eliminates the dependence of detection calling to identify negative probesets. In the present study, we compared PVAC filtering with all-absent probesets as negative probesets (PVAC_AAB) to PVAC filtering with anti-genomic probesets as negative probesets (PVAC_AG; only for HG-U219 arrays). A detailed discussion of probeset filtering strategies is given in the S1 File. The list of probesets remained after PVAC filtering (PVAC_AAB and PVAC_AG) in the matched datasets are given in the S3 and S4 Tables.

### Module score computation and calculation of module score correlation

#### Module score algorithm

Typically, a module score is computed as a weighted average, where the weights are +/-1 depending on the direction of the association of an individual gene to the biological signal interrogated [9,24], such as mutation status, pathway activation level, survival benefit and resistance to therapy. In this analysis, a modified version of the above method has been used to simplify the interpretation of modules scores. Specifically, we averaged the expression of positively and negatively associated genes independently and computed their differences. With this modified algorithm, a positive module score implies that the average expression of positively associated genes is higher than the average expression of negatively associated genes and vice versa. The above interpretation of module scores is possible with the unmodified algorithm only if the proportion of positively and negatively associated genes per gene-module is equal, but this is not always true. A comparison between module score computation in log2 and linear scale gene expression values revealed an improvement in module score correlation profile when expression values are in log2 scale (S5 and S15 Figs). Hence in the present study module scores were computed in log2 scale expression values. Note that, for the above interpretation of module scores to hold all log2 scale gene expression values should be positive (i.e., linear scale expression should be higher than 1), which was the case in our matched dataset.

#### Module score correlation

There were five matched datasets used in this analysis, and each dataset contains eight samples (see the "Experimental design and samples details" section). The correlation coefficient of module scores between each reference datasets and their respective relevant matched datasets was computed as follows. (1) From the total of eight samples, all 7-sample combinations were selected. (2) From these different sets of 7-sample combinations, the reference to matched datasets module-score correlation coefficient was computed for each gene-module. (3) The average of correlation coefficient computed from all the 7-sample combinations was used as the correlation coefficient in this analysis. This average correlation coefficient can be considered as a robust measure of module score agreement.

### Definition of gene-module reproducibility

Using the Spearman correlation coefficient (see the “Module score correlation” section) we measured the agreement of the module scores between the reference and the matching test datasets. A high correlation represents a good agreement between the reference and the test module score (i.e., the majority of the up/down-regulated genes in the reference dataset are also up/down-regulated in the matched datasets). It is entirely possible that a gene-module with all genes down-regulated or absent in the reference dataset gets a high module score correlation (S6 Fig). Hence a high module score correlation cannot be used to distinguish signals that are present or absent in matched datasets, instead, it merely gives a measure of the agreement of the module score between the reference dataset and the matched datasets, which we call reproducibility. If there exists a high correlation (agreement) between module scores from reference and matched datasets, we consider that the gene-modules derived from the reference expression profiles are reproducible in matched expression profiles and vice versa. An arbitrary cutoff of correlation coefficient greater than 0.8 was used to distinguish reproducible biological signals from non-reproducible ones. In this analysis, we did not distinguish between biological signals which are present or absent in the datasets. We only looked into the agreement in module score based biological signal, by correlating module scores computed from reference and matched datasets.

### External breast cancer gene-modules considered

Two sets of external breast cancer gene-modules were considered in this analysis: the 1^st^ set contains 76 BC gene-modules manually extracted from original publications (external-module-set1), and the 2^nd^ set contains 541 BC gene-modules extracted from the GeneSigDB version-4 [25] (external-module-set2). We defined the full gene-module (full-module) as the subset of the original module genes which are present in both the HG-U133plus2 and HG-U219 arrays. For each of these full-modules, a module-subset was derived for each matched dataset, by discarding genes from the full-modules which were not present after PVAC filtering (PVAC_AAB/PVAC_AG). This method gave five different versions of the module-subsets for each full-module, as PVAC filtering is dataset dependent and yields five different sets of PVAC selected genes from the five matched datasets. Note that the two versions of PVAC filtering detailed in “Probe set filtering” section were used (PVAC_AAB/PVAC_AG) and hence for each full module ten versions of module-subsets were generated. If any of the module-subset from any matched dataset contains less than two genes in it, then all versions of that module were discarded from the analysis. Each of the remaining module-subsets was validated by correlating the full-module score to the respective module-subset score computed from the gold standard expression dataset derived from FF RNA using the HG-U133plus2 array with the 3’IVT kit (u133p2.3ivt.ff). Both full and subset modules were discarded from the analysis if any version of the module-subset had a Spearman correlation less than 0.9 to its corresponding full-module. The correlation values of module-subsets from the u133p2.3ivt.ffpe dataset was not considered for the above validation because of close to background expression values in this dataset. After validation, the external-module-set1 contained 37 (~49%) gene-modules and the external-module-set2 contained 108 (~20%) gene-modules. References of the external gene-modules considered are given in the S5 Table.

### Control gene modules

Although the major subtypes of breast cancer have been included in this study, biological signals represented by some gene modules might be absent from all samples in the dataset, such as gene-modules representing TP53 mutation status. To assess the impact of signals which are absent from all samples in the analysis, a set of manually curated tissue-specific gene modules from Dezso et al. 2008 [26], which are known to be absent in breast tissue (negative controls), have been included in the analysis as control modules. The above set of control modules also include a breast tissue specific gene module and housekeeping (genes which are up-regulated in all tissues considered in Dezso et al.) gene module. These two gene-modules can be considered as positive controls as they are typically present in every breast tissue. Besides, the set of common housekeeping genes present in HG-U219 and HG-U133plus2 arrays were combined as a positive control gene module, and this was included along with the set of control modules from Dezso et al. [26]. Since the genes in the control modules are all up-regulated in their respective tissues, we assigned a weight of +1 for all genes in each control module. Note that majority of these control modules did not pass the correlation filtering mentioned in the above section. Hence all control modules which passed size filter (n> = 2) were used in this study.

### De-novo ER/HER2 gene-module development

ER/HER2 immunohistochemistry (IHC) status based differentially expressed genes were identified from each matched dataset using both fold change and Welch's t-test in log2 scale expression data [27]. Similar to the MAQC-I approach [28], genes with absolute fold change > 2 and Welch's t-test p-value < 0.05, were grouped into gene-modules. ROC analysis was used to validate these de-novo signatures in the external datasets for which ER/HER2 IHC status was available.

### External breast cancer datasets used

Expression datasets derived from frozen samples [11] and FFPE samples [10], for which ER/HER2 IHC status was available, were extracted from GEO [18] using the accession numbers GSE20713 and GSE53031, respectively. The normalized expression datasets (RMA background corrected, quantile normalized and median-polish summarized) available from GEO, were used in this study. Details of detection calling and PVAC filtering on these datasets were given in the S1 File.

### Statistical analysis

The comparisons were performed either by using box-and-whisker plots and or by Spearman's rank correlation coefficient. Log2 scale data was used for module-score computation and differential expression analysis. All analysis were performed in R statistical software [15], version:3.1.1 using the following R packages affy [17], reshape2 [29], pROC [30], plotROC [31]. ggplot2 [32], grid [15] colortools [33], RColorBrewer [34], and gridExtra [35].

## Results

### An unrealistic low background is observed in the HG-U219 arrays using the ovation kit

**Fig 1:**
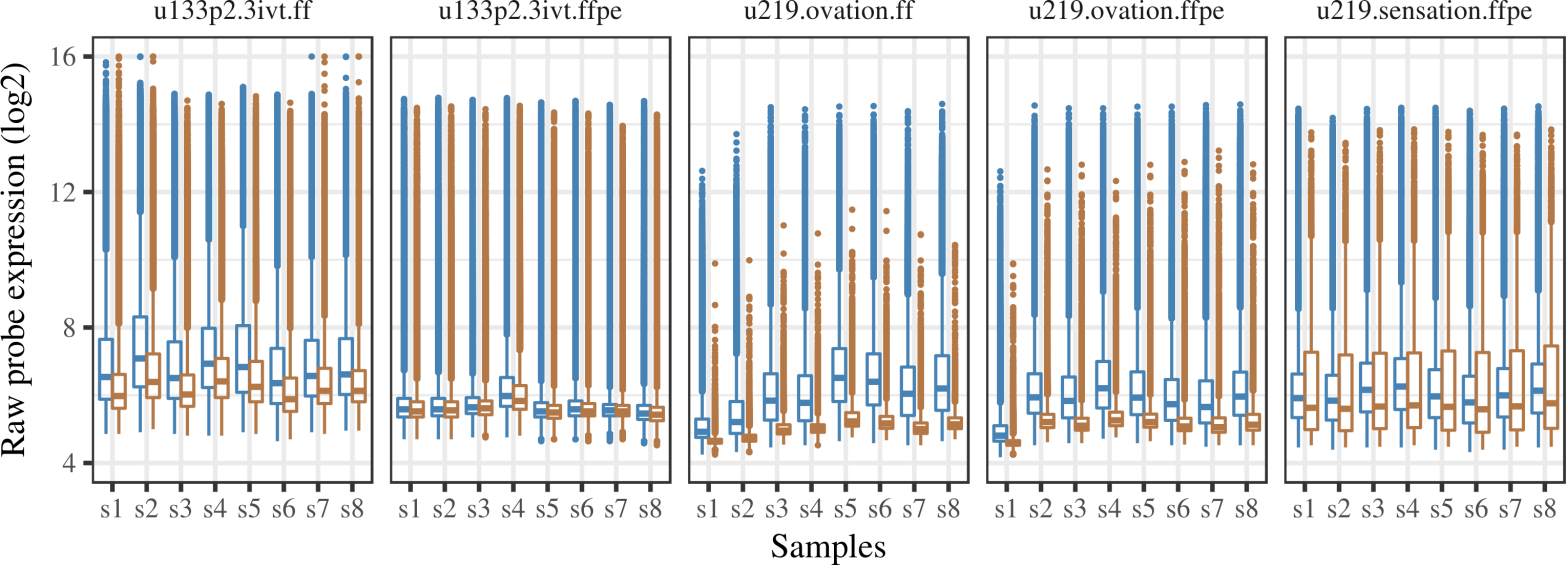
Raw expression distribution of the genomic and the background probes. Blue represents the genomic probes (PM probes), and brown represents the background probes (MM/anti-genomic probes).

A higher background is expected in the HG-U219 array compared to the HG-U133plus2 array, as the potential for GC content based cross-hybridization is higher [36,37] in the HG-U219's anti-genomic background probes compared to the entire MM probes in the HG-U133plus2 array. However, the HG-U219 arrays using the ovation kit (hgu219.ovation.ffpe/ff) resulted in a low background whereas using the sensation kit (hgu219.sensation.ffpe) we observed a higher background compared to the HG-U133plus2 array, as expected (Fig 1). If the low background expression in the datasets using the ovation kit was true, then the entire genes in the array should be called present; but this is highly unlikely. Hence we considered this low background as unrealistic. Further, the raw expression values from the u133p2.3ivt.ffpe dataset was close to background, which is expected as the polyA-tail dependent 3ivt kit is not ideal for amplifying degraded RNA. Although not comparable to the other matched datasets, we kept this dataset in the analysis to see if some signals could still be recovered from it. The expression of positive genomic controls was well above background in all datasets except in the u133p2.3ivt.ffpe dataset, in which the expression of positive controls and its corresponding background expression were slightly mixed up (S3 Fig).

### FFPE derived expression profiles are not at par with the matching frozen derived expression profiles

#### PVAC filtering

Among the 17239 genes in common between the HG-U133plus2 and HG-U219 arrays (matched using the HUGO gene symbol available from the respective NetAffx array annotation files [38], version 34), the number of PVAC selected unique genes reflects the effect of degraded RNA in FFPE samples (see S3 and S4 Tables). PVAC_AAB identified 11882 (69%) and 7337 (43%) reliable genes from the frozen datasets (u133p2.3ivt.ff and u219.ovation.ff, respectively) whereas only 2056 (12%), 4247 (25%) and 4474 (26%) genes were identified from the FFPE datasets (u133p2.3ivt.ffpe, u219.ovation.ffpe and u219.sensation.ffpe, respectively). PVAC_AG identified roughly double probesets than PVAC_AAB from HG-U219 arrays with Nugen’s ovation kit (11930 (69%) from u219.ovation.ff and 8613 (50%) from u219.ovation.ffpe). Note that in these HG_U219 datasets with ovation kits, detection calling was compromised due to its unexpectedly low background and this could be the reason for the reduced number of reliable probesets from these datasets with PVAC_AAB as PVAC_AAB depends on detection calling to identify negative probesets. It is intriguing that whether the additional set of genes identified by PVAC_AG compared to PVAC_AAB from HG-U219 arrays with ovation kit measure reliable information, as it is clear that the additional set of genes identified by PAVC_AG from these datasets is due to the unexpected low expression levels of anti-genomic probesets (which are used as negative probesets for PVAC filtering) in these arrays. It is noteworthy that both PVAC_AAB and PVAC_AG identified a similar proportion of genes as reliable from the FFPE dataset generated using HG-U219 arrays with sensation kit, in which the expression levels of anti-genomic probesets (background) behaves as expected. Altogether, the number of reliable genes identified from FFPE datasets by the two PVAC filtering strategies were low compared to that identified from their respective frozen datasets.

#### Gene correlations

**Fig 2:**
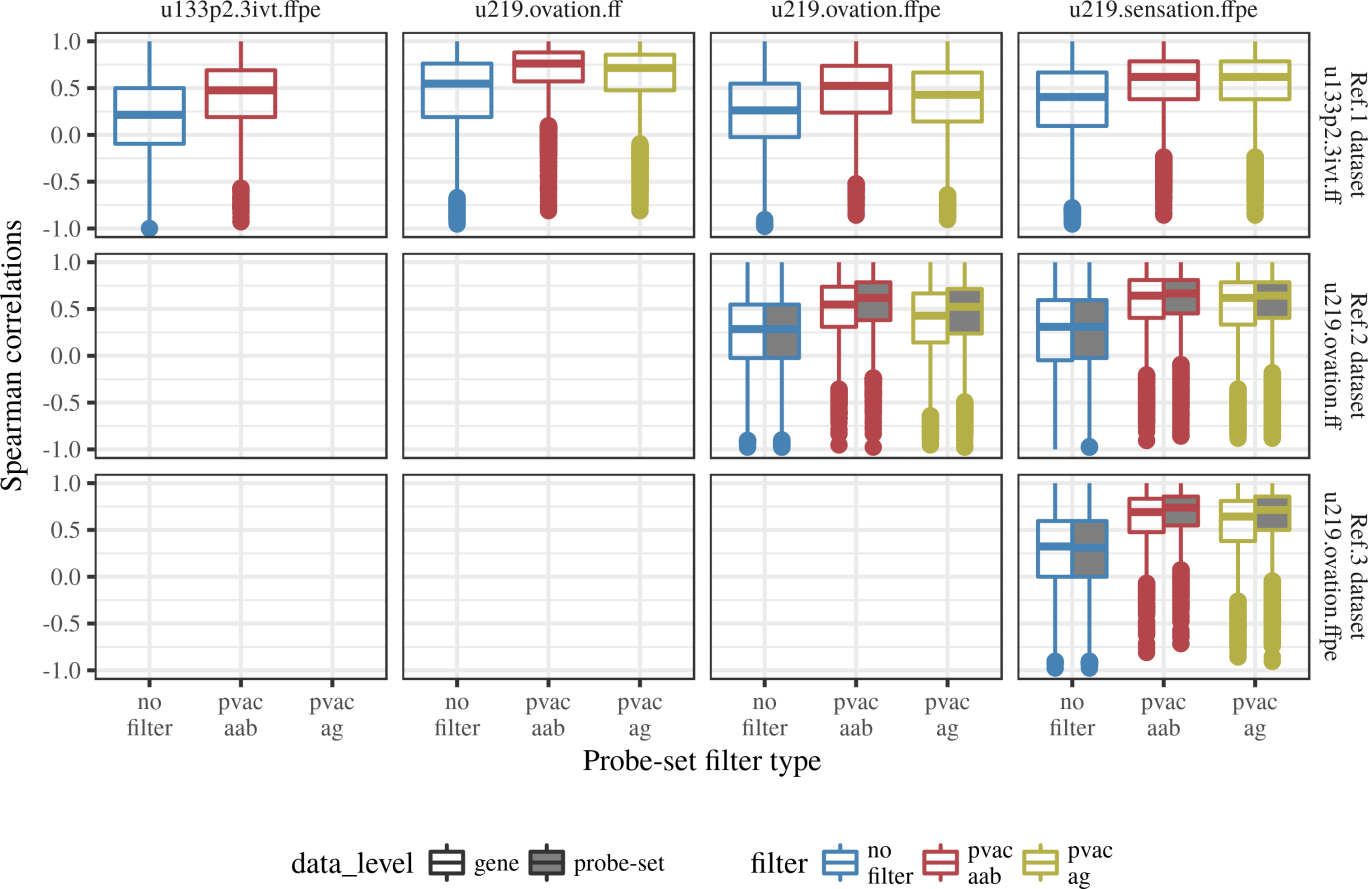
Gene correlations between reference datasets and matched datasets. Blue, red and green color represents the distribution of gene correlations before filtering, after PVAC_AAB filtering and after PVAC_AG filtering respectively. Boxplots filled in gray color represents the distribution of probeset correlations.

In ref-1 comparisons, the two frozen datasets (the reference hgu133p2.3ivt.ff and hgu219.ovation.ff) showed the best relative gene correlations (Fig 2); ~50% of genes have correlations above 0.75 (the percentage is computed using the per dataset common genes between the reference and matched datasets). The percentage of highly correlated genes was reduced to ~25% when comparing the gene expression profiles between reference frozen and matching FFPE datasets in both ref-1 and ref-2 comparisons (Fig 2). In ref-3 comparison between FFPE datasets (u219.ovation.ffpe and u219.sensation.ffpe) ~25% of genes have correlation above 0.75, which is similar to comparisons between frozen and FFPE datasets in ref-1 and ref-2 (Fig 2). Note that the probeset level correlation among HG-U219 datasets was similar to corresponding gene level correlations. This probeset level analysis is not performed in ref-1 comparisons due to different arrays included in the ref-1 comparison. Further, we observed an overall low correlation in the relative gene expression between the frozen reference dataset and all the matched FFPE datasets, whereas the correlation was higher in the matched frozen dataset (Fig 2). Collectively, this implies that FFPE expression profiles are not at par with the matching frozen expression profiles.

#### Sample correlations

A strong influence of both the amplification-and-labeling kit and the sample preservation method was observed in the sample correlations between the reference and matched datasets (S4 Fig), which is expected as we correlated datasets which used different amplification-and-labeling kits. Also, it is well known that the absolute expression value of a gene is significantly affected by the kit used [39,40]. The sample correlations were improved when probeset level expression data is used compared to gene-level expression data in ref-2 and ref-3 comparisons. The low sample correlation at gene level is due to the maximum variant probeset criteria used to select a single probeset among the multiple probesets representing a gene. So a gene could potentially be represented by different probesets in different datasets, which in turn yield a low sample correlation.

### Not all external gene-modules are valid in the FFPE derived gene expression profiles

Gene-module based biological signals are mainly used for classifying patients in the clinics (e.g., proliferation signal, PI3K pathway activation level, and TP53 mutation status). The genes up-regulated in one phenotype are generally down-regulated in the other phenotypes. The classification potential of each gene-module gets compromised if either the relevant genes are degraded due to the fixation process or the FFPE specific sample preparation kits failed to recover them. The module score correlation coefficient between the reference and the matching test datasets measures the agreement in the classification potential of the respective gene-module between the reference and the matched dataset considered. A high correlation(agreement) of the modules score computed from the reference dataset to the matched dataset could imply that the gene-modules represent a similar biological signal in both the datasets. Hence using the matched FFPE/frozen datasets, we tested whether the external gene-modules (mainly derived from frozen expression profiles) are valid in FFPE expression profiles. Since some genes in the FFPE expression profiles may not be as reliable as those from the matching frozen expression profiles due to RNA degradation, for each external gene-module, a module-subset was also defined per matched dataset (n = 5) such that the five module-subsets contain only individual dataset specific PVAC selected genes from the full gene-module. Since we used two types of PVAC filtering (PVAC_AAB and PVAC_AG), altogether there were ten module-subsets per each full module. Considering the full-module scores from the frozen reference dataset as the truth, we correlated the module scores of both full-modules and their corresponding module-subsets computed from each matched dataset to reference module scores (see “Module score correlation” section). The correlation values are given in the S6 and S7 Tables.

**Fig 3:**
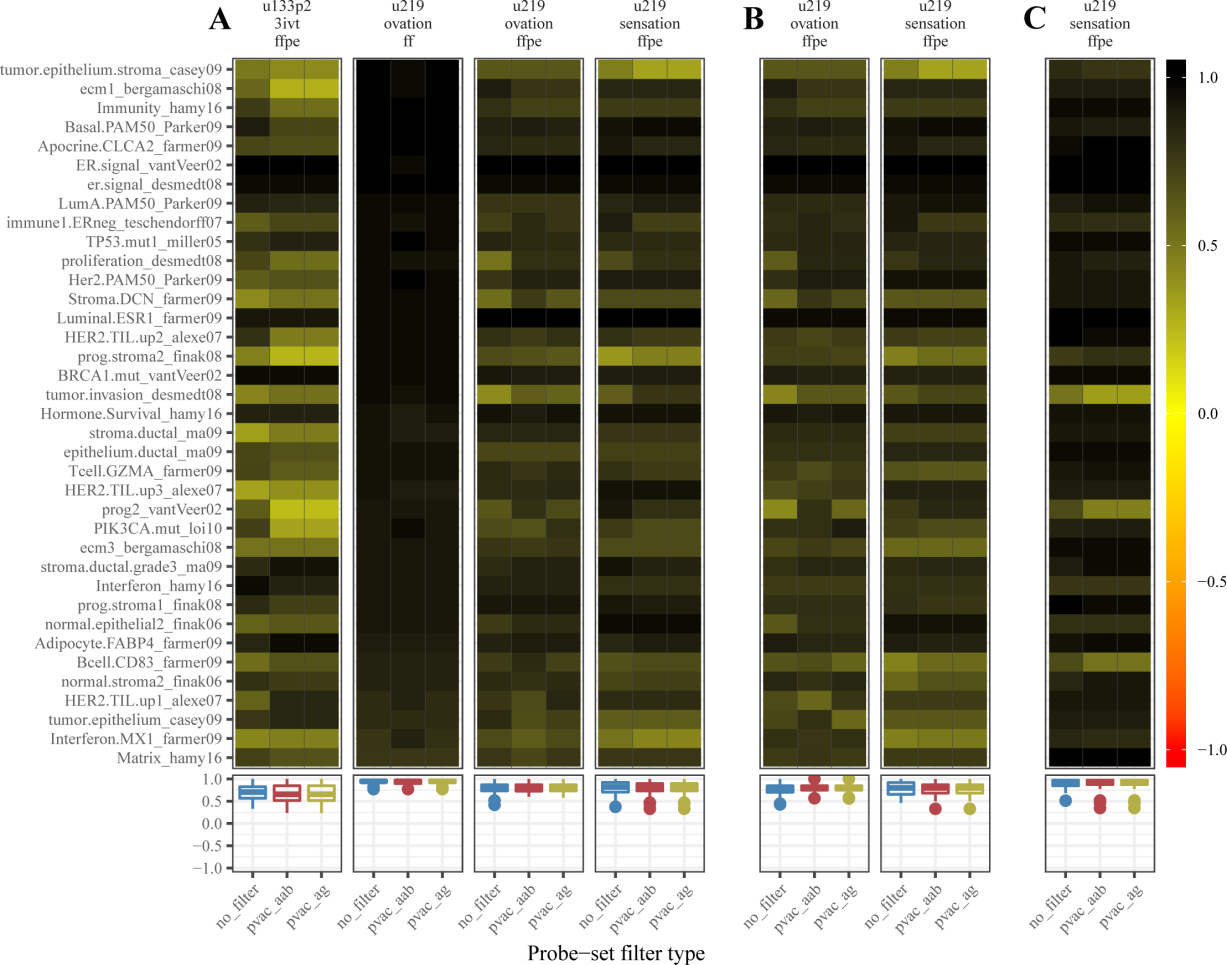
Correlation profile of the external-module-set1 gene-modules in the matched datasets. Correlation of the full-module scores of the external-module-set1 gene-modules (manually extracted from literature) computed from ref-1/ref-2/ref-3 reference dataset to the respective full-module/module-subset scores computed from the matched datasets. The panel A contains the heat map of the correlation profiles from the ref-1 comparison where the reference dataset was u133p2.3ivt.ff, and panel B and C contains similar plots from ref-2 and ref-3 comparisons. The reference datasets in ref-2 and ref-3 comparisons were hgu219.ovation.ff and hgu219.ovation.ffpe respectively. An arbitrary cutoff of correlation coefficient equal to 0.8 was used to distinguish a gene-module as concordant/reproducible or discordant/non-reproducible. The blue, red, and green colored boxplots beneath the heat map represent the distribution of module-score correlations before filtering, after PVAC_aab filtering and after PVAC_ag filtering respectively.

We found that the dataset-specific module scores of both full-modules and their corresponding module-subsets (based on PVAC_AAB and PVAC_AG) were similarly correlated to the reference module scores in all ref-1/ref-2/ref-3 comparisons (Figs 3 and S5). In ref-1 comparisons, the two frozen datasets (the reference hgu133p2.3ivt.ff and the matched hgu219.ovation.ff) showed the best relative module-score correlations (Figs 3 and S5); 95% of external-module-set1 and 75% of external-module-set2 modules have correlations above 0.8. The percentage of highly correlated (rho > 0.8) gene-modules between frozen datasets in ref-1 comparison was reduced to 1) ~ 43–51% in external-module-set1, and 2) to ~35–38% in external-module-set2 when full module-scores of reference frozen datasets were correlated to full module-scores of matching HG-U219 FFPE datasets in both ref-1 and ref-2 comparisons (Figs 3 and S5 and S6 and S7 Tables). Of note around 30% of external-module-set1 and 17% of external-module-set2 gene-modules from the u133p2.3ivt.ffpe dataset was highly correlated to the reference frozen u133p2.3ivt.ff dataset in ref-1 comparison, suggesting that some reliable signals could still be recovered from FFPE RNA with 3’IVT kit (Figs 3 and S5 and S6 and S7 Tables). Finally, in ref-3 comparison between the FFPE datasets u219.ovation.ffpe and u219.sensation.ffpe, the module scores of external-module-set1/set2 were highly correlated; 83% of external-module set1 and 72% of external-module-set2 with rho > 0.8 (Figs 3 and S5), This percentage is similar to the percentage of highly correlated modules between frozen datasets in ref-1 comparison. Altogether the three types of comparisons suggest that when moving from FF RNA to FFPE RNA for microarray expression profiling almost half of the signals that may potentially present in frozen samples could be lost, given that the signals extracted from FF RNA are the 100% truth. However, the high correlation between matched FFPE HG-U219 datasets with ovation and sensation kit suggests that within the FFPE realm the signals were reproducible (concordant), similar to that is seen within the FF realm independent of the amplification kit used.

Among the biological signals represented by the control gene modules, which are supposed to be absent in breast tissue, we observed a high correlation between module scores computed from the reference dataset and the matched frozen dataset in ref-1 comparison, suggesting that a high correlation may not imply the presence or the absence of a biological signal. Instead, it measures merely the agreement of the module score between the reference and the test datasets (S6 Fig and S8 Table). Further, we observe that the module score correlations were influenced by the highly expressed genes within the module. Specifically, we correlated module scores computed from reference dataset to that computed from matched datasets using PVAC_AAB/PVAC_AG modules-subsets and to a similar set of module-subsets derived by grouping genes in the full module which are not selected by PVAC_AAB/PVAC_AG (~PVAC_AAB and ~PVAC_AG). We observed that PVAC_AAB/PVAC_AG module-subsets were highly correlated to reference full modules, whereas ~PVAC_AAB / ~PVAC_AG module-subset correlation with reference full modules was low (S7 Fig). Of note PVAC filtering preferentially selects highly expressed probesets within each dataset (S8 Fig).

In the same line, we also generated module-subsets from full modules based on the dataset-specific mean expression distribution of module genes. Module-subsets were generated by grouping genes that fall into a single quartile based on the above distribution. With this approach four module-subsets (q1, q2, q3 and q4) per full module were generated such that the average expression of genes per module-subsets is in the order q4 > q3 > q2 > q1. The module-score correlation profiles of these module-subsets with full module were investigated. We were expected to see a systematic increase in the magnitude of the correlation profile from q1 to q4 when these module-subsets were correlated to full reference modules. Although we observe this trend in some datasets, this was not consistent among different matched datasets (S7 Fig), suggesting that the high expression of module genes not be the only criteria that influence reference to matched datasets module score correlations.

To investigate further, we computed gene module quality metrics and plotted these against the reference to matched dataset module score correlations. The following metrics were considered; 1) average expression of genes per module per dataset to identify any potential influence of module gene expression levels on module correlation, 2) average pair-wise correlation of genes per module per dataset to identify any potential influence of module gene’s co-expression levels (or in other words module genes coherence) on module correlation. We observed that when module coherence or average expression increases the respective module correlations were also increased in external-module-set2, but this trend was reversed when external-module-set1 was used (S9 and S10 Figs). This reversal of trend between external-module-set1 and external-module-set2 was due to the influence of module-score computation algorithm on module score correlation (S11 and S12 Figs).

### Reliable gene-modules can be developed using FFPE gene expression datasets

To test the reliability of the gene-modules developed from the FFPE datasets, we recreated already established ER and HER2 pathway gene-modules from all matched datasets. The percentage of genes shared among different versions of the de-novo ER and HER2 gene modules developed was not high (S13 Fig). Further, the gene-modules derived from both non-filtered and PVAC filtered (PVAC_AAB and PVAC_AG) datasets contained a similar number of genes, and almost all genes of the gene-modules derived from the PVAC filtered dataset were present in the gene-modules derived from the non-filtered datasets (S13 Fig).

**Fig 4:**
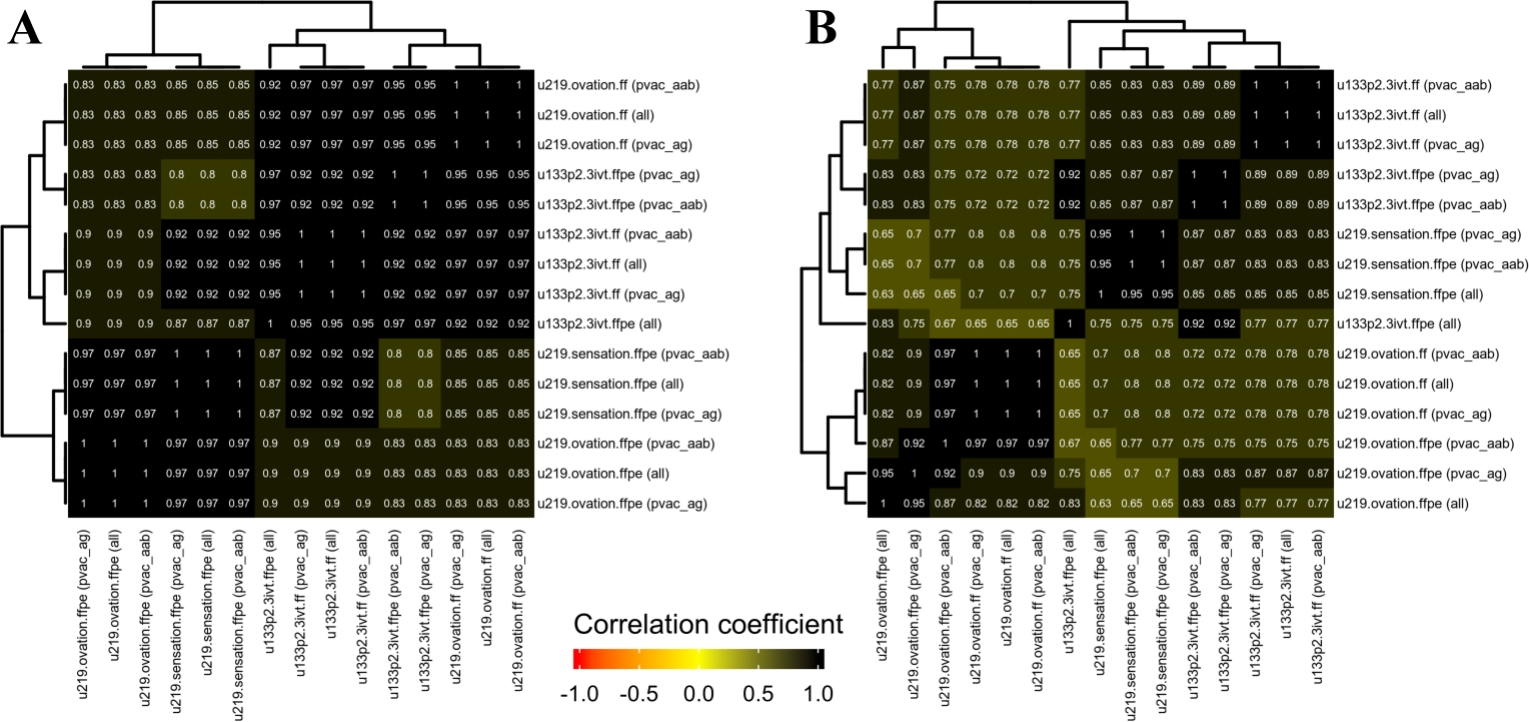
Heatmap of the correlation matrix computed from the de-novo ER/HER2 gene-modules. A) Correlation between the different versions of the de-novo ER gene-module scores computed from the respectively matched dataset used to derive it. B) Correlation between the different versions of the de-novo HER2 gene-module scores computed from the respectively matched dataset used to derive it.

To see if the different versions of the de novo ER/HER2 gene-modules extracted similar information from the respective datasets used to derive them, we correlated the module scores of all de novo ER/HER2 gene-modules to each other (Fig 4). De-novo ER modules derived from all versions of matched datasets (non-filtered, PVAC_AAB filtered, and PVAC_AG filtered) were all highly correlated to each other, implying that the ER signal extracted from both frozen and FFPE datasets was similar irrespective of the array/amplification-and-labeling kit used. A similar trend was observed for the de-novo HER2 modules derived from matched datasets except for de-novo HER2 modules derived from the non-filtered versions of the three FFPE datasets; u133p2.3ivt.ffpe, u219.ovation.ffpe and u219.sensation.ffpe. Of note, the de-novo HER2 module from non-filtered u219.ovation.ffpe dataset was not highly correlated to any versions of de-novo HER2 modules from u219.sensation.ffpe and vice-versa. However, the de-novo modules from PVAC filtered versions of all three FFPE datasets were correlated well with each other and to the de-novo HER2 modules from FF datasets (Fig 4).

**Fig 5:**
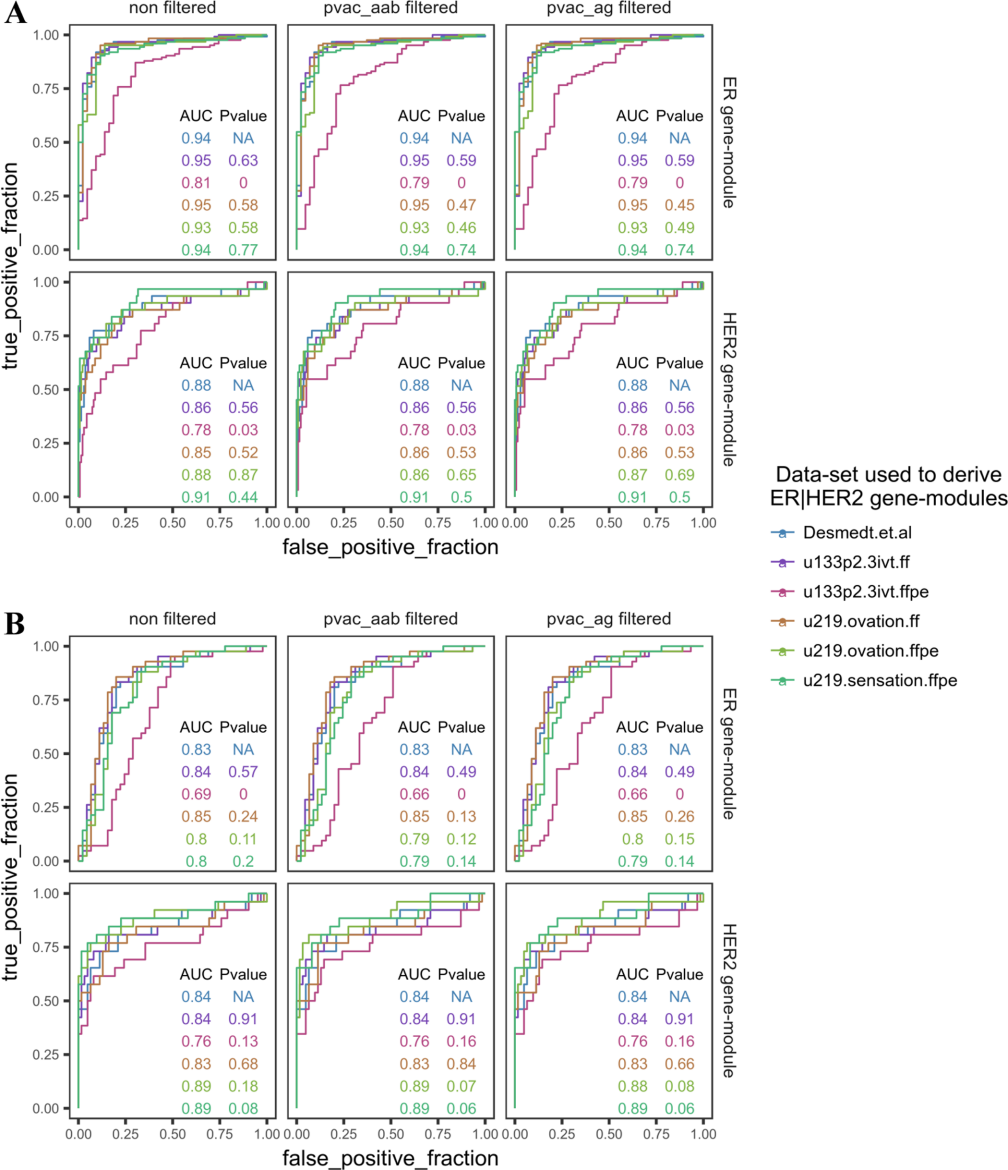
Validating the de novo ER/HER2 gene-modules in the independent datasets. Validating the de-novo ER/HER2 gene-modules derived from each matched dataset in the independent FFPE expression dataset from Azim et al. 2014 (A), and frozen expression dataset from Dedeurwaerder et al. 2011 (B). P-value is calculated using DeLong's test for correlated ROC curves where the reference ROC curves were generated using the respective ER/HER2 gene-module derived from Desmedt et al. 2008.

Using publicly available breast cancer expression datasets profiled using frozen [11] and FFPE [10] samples with known ER and HER2 status, we evaluated the different versions of the de-novo ER/HER2 gene-modules along with the ER/HER2 gene-modules from Desmedt et al. [9] using ROC analysis (Fig 5). Except for the de-novo ER/HER2 modules derived from all versions of the u133p2.3ivt.ffpe dataset, all other de-novo modules have comparable classification potential similar to Desmedt et al.’s ER/HER2 gene modules (The classification potential is measured as the area under the ROC curve; AUC). The de-novo ER/HER2 modules from u133p2.3ivt.ffpe datasets showed significantly low classification potential in the two validation datasets. Further, the de-novo modules derived from PVAC filtered (PVAC_AAB/PVAC_AG) and non-filtered datasets showed similar AUC within each validation datasets.

## Discussion

Due to the commercial availability of FFPE specific RNA amplification and labeling kits, large-scale gene expression profiling from archival FFPE samples is currently feasible. However, studies which profiled FFPE samples reported a high range of percent-present values [1–6], reflecting the varying proportion of reliable genes in FFPE expression profiles. The high range of percent-present values reported may pose a problem in the differential expression analysis due to the spurious association introduced by inconsistent RNA degradation. An obvious solution to control this problem is to introduce a probe set filtering step before the differential expression analysis. Among the different filtering strategies considered, only the PVAC [22] method seems to be well suited for FFPE derived expression profiles. Although Calleri et al. have shown improvement in concordance between expression profiles derived from FF and FFPE RNA using an alternative CDF for HG-U133plus array [41], we prefer to seek strategies for improved analysis with standard CDF and hence did not consider an alternative CDF based analysis. Further, the probeset filtering strategy used in this study could get affected by the varying probeset size in alternative CDF.

By introducing the probe set filtering step we were expected to see, 1) a subset of genes from the matched datasets which are highly correlated with genes from the reference dataset, 2) a better performance of the gene-modules developed from the probeset filtered dataset and 3) an improvement in the correlation of external gene-modules between FFPE expression datasets and the reference dataset. As expected, the PVAC selected gene subsets contained a significant proportion of genes which are better correlated with genes from the reference dataset compared to the entire genes in the array, but they still contained genes which are poorly correlated (rho: -1 to 0.5) to the reference gene expression (Fig 2). However, the PVAC filtering didn’t seem to improve, 1) the performance of the de novo ER/HER2 gene-modules (based on differential expression analysis) derived from the FFPE expression profiles (Figs 4 and 5) and 2) the correlation profile of external gene-modules between the reference dataset and the matched datasets (Figs 3 and S5). PVAC’s failure to improve results could be because PVAC preferentially selects highly expressed probesets among the datasets and the module-score correlation between matched datasets is influenced by the highly expressed genes within a gene module (S7, S9, and S10 Figs). Hence if a full module is highly correlated between matched datasets, its corresponding sub-modules derived based on PVAC filtering is likely to be highly correlated between the same pair of matched datasets as the sub-module retains the highly expressed genes from the full module. In short, PVAC filtering seems to be redundant in our analysis. Because if there exists a significant proportion of highly expressed genes in a full module, enough to capture the biological signal, the full module is likely to capture it even if it contains a set of poorly expressed (possibly due to RNA degradation) genes representing the same pathway/biological signal. Also, the de-novo ER/HER2 modules derived from non-filtered and PVAC_AAB/PVAC_AG filtered dataset contains a similar number of differentially expressed genes and almost all genes identified from the PVAC filtered dataset were present in the de-novo module from the corresponding non-filtered dataset (S13 Fig). The congruence in the de-novo module gene list from different versions of a single dataset suggests that the differential expression analysis algorithm used in this study mainly identifies the highly expressed genes and discards the potentially degraded / low expressed genes. Collectively, PVAC probeset filtering seems to be a redundant procedure in FF/FFPE microarray expression analysis. It should be noted that the present study failed to reproduce the high range of PP values reported in other studies which profiled FFPE derived RNA (S14 Fig) [1–6]. Hence the comparison between non-filtered and PVAC filtered datasets was compromised to an extent. However we did not observe any reduction in the reliability of analysis performed in PVAC filtered dataset compared to the non-filtered dataset, and PVAC filtering is suitable for FFPE expression profiles as it considers the probe level expression consistency. Hence PVAC probe set filtering can be integrated into FF/FFPE microarray gene expression analysis, as it successfully retains reliable probesets for downstream analysis.

By recreating gene modules of two prominent biological signals present in breast cancer, ER and HER2, we have demonstrated that if the gene-modules derived from FFPE samples represents an incompletely recovered biological signal, such as the ER/HER2 de-novo modules extracted from u133p2.3ivt.ffpe, it is unlikely to be reproducible in external datasets (Figs 4 and 5). Further, the FFPE specific sensation and ovation kit successfully recovered reproducible ER signal and HER2 signal from FFPE RNA.

We have also seen that the expression profiles derived from matching FFPE and FF samples were not equivalent. Almost 50% of the gene modules that are highly correlated between matching FF datasets were not highly correlated to FFPE datasets (ref-1 and ref2 comparisons). However, the high correlation between the matched FFPE HG-U219 datasets with ovation and sensation kit (ref-3 comparison) suggests that within the FFPE realm the signals were reproducible (concordant); similar to what is seen within the FF realm (ref-1 comparison). Altogether the external module validation analysis implies that, when moving from FF RNA to FFPE RNA in microarray expression profiling, almost half of the signals that may potentially present in FF RNA could be lost, considering the signals extracted from FF RNA is the 100% truth. However, once in FFPE realm, the signals seem to be correlated between matched datasets independent of amplification-and-labeling kit used (Figs 3 and S5).

The FFPE specific amplification and labeling kits (random + dT primer based) considered in our analysis succeeded in extracting more biological signal from FFPE derived RNAs that are highly correlated to respective signals from FF RNA (~50%), than the dT primer based 3'IVT kit (~20%). However, the FFPE kits failed to extract all biological signal from FFPE RNA to make the biological signal concordance between matched FF and FFPE datasets 100%. Of note, around 20% biological signals that are highly correlated to matching FF samples, could still be recovered from FFPE samples using dT primer based 3’IVT kit (Figs 3 and S5).

Altogether our analysis implies that a part of the biological signal that can be extracted from FF RNA using appropriate protocols, may not be transferred to the FFPE realm and vice versa. Hence expression profiles derived from FF RNA and FFPE RNA may not be considered equivalent and improved sample preparation protocols are required to reduce this discordance in expression profiles derived from matching FF and FFPE samples. Our analysis has limitations, we did not attempt to check the biological meaning of external modules considered in our analysis, nor we checked whether they are present in the datasets.

We have seen in this study that the microarray sample preparation protocol has a profound influence on the recovery of a biological signal from degraded FFPE RNA. By eliminating the sample preparation step required in the microarray technology (i.e., cDNA synthesis, amplification, and labeling), the nCounter technology effectively reduces the bias introduced by the sample preparation step [42]. However, a whole genome approach is currently not feasible with nCounter technology as it supports only a maximum of 800 probe pairs (i.e., reporter probe and capture probe) in a single multiplexed experiment [43]. Considering the theoretically unlimited dynamic range of RNAseq expression measurements and the non-requirement of pre-designed probes used in hybridization-based technologies [44], RNAseq seems to recover the biological signals from FFPE RNA that cannot be recovered using hybridization-based technologies, such as microarrays and NanoString. Moreover, Li et al. have shown that comparable results could be obtained using RNAseq from FF and FFPE derived RNA [45], which is promising. In our study, we took an unbiased approach in investigating the signal recovery rate between microarray expression profiles derived from matching FF and FFPE RNA, by checking the concordance rate of all published breast cancer gene-modules extracted from geneSigDB. We found that among the signals (gene-modules) compared almost half of the signals are concordant between FF and FFPE derived matching expression profiles. The remaining half of the signal, which are discordant between FF and FFPE expression profiles, seems to be concordant among matching FFPE derived expression profiles, and among matching FF derived expression profiles when the RNAs are pre-processed with appropriate FF/FFPE specific protocols. The question of whether RNAseq could recover biological signals from FFPE RNA to make a 100% concordance to signals recovered using RNAseq/microarrays from matching FF RNA, which may not be possible with microarrays at present, remains unanswered. However, results from our study would be useful for researchers performing gene expression profiling from FFPE derived RNA.

In conclusion, the two FFPE specific amplification-and-labeling kits from Affymetrix and Nugen performed similarly, and successfully recovered much more biological signals from FFPE RNA which are highly correlated to respective signals from FF RNA, than the 3'IVT kit. However, these FFPE specific kits failed to extract all biological signal from FFPE RNA to make the biological signal concordance between matched FF and FFPE RNA 100%. Further, when moving from FF RNA to FFPE RNA in microarray expression profiling, almost half of the signals that may potentially present in FF RNA could be lost, given that the signals extracted from FF RNA are the 100% truth. However, once in FFPE realm, the signals seem to be correlated between matched FFPE datasets independent of amplification-and-labeling kit used. The FFPE specific ovation and sensation kits used in this study successfully recovered reproducible ER and HER2 gene module FFPE expression profiles. Although PVAC probeset filtering did not seem to improve the overall results in this study, it successfully eliminates a significant proportion of unreliable probesets from FFPE/frozen expression profiles, hence limiting the analysis to PVAC selected probesets would be advantageous in FFPE expression profiling studies. Further, our analysis highlighted that microarrays still requires more optimized amplification-and-sample-preparation protocols to improve the biological signal concordance between expression profiles derived from FF and FFPE RNA.

## Supporting information

S1 Fig Experiment design. (EPS)

S2 Fig Dataset comparison structure. (EPS)

S3 Fig Raw expression distribution of the positive genomic controls and the background probes. Blue color represents the raw PM probe expression of the 100 positive genomic control genes, and brown color represents the raw background expression. The background expression of the HG-U133plus2 array constitutes the MM probe expression of the 100 genomic positive control genes, and that of the HG-U219 array constitutes the expression of the 23 anti-genomic probesets.
(TIFF)

S4 Fig Sample correlation between reference datasets and matched datasets. Blue, red and green color points and lines represent the distribution of sample correlations before filtering, after PVAC_aab filtering and after PVAC_ag filtering respectively. The open circle represents the distribution of sample correlation at the probeset level. The correlation coefficient is computed using common genes/probesets between reference datasets and matched datasets.
(TIFF)

S5 Fig Correlation profile of the external-module-set2 gene-modules in the matched datasets (log2 scale). Module-score were computed from log2 scale expression data. Correlation of the full-module scores of the external-module-set2 gene-modules (geneSigDB) computed from ref-1/ref-2/ref-3 reference dataset to the respective full-module/module-subset scores computed from the matched datasets. The panel A contains the heat map of the correlation profiles from the ref-1 comparison where the reference dataset was u133p2.3ivt.ff, and panel B and C contains similar plots from ref-2 and ref-3 comparisons. The reference datasets in ref-2 and ref-3 comparisons were hgu219.ovation.ff and hgu219.ovation.ffpe respectively. An arbitrary cutoff of correlation coefficient equal to 0.8 was used to distinguish a gene-module as concordant/reproducible or discordant/non-reproducible. The blue, red, and green colored boxplots beneath the heat map represent the distribution of module-score correlations before filtering, after PVAC_aab filtering and after PVAC_ag filtering respectively.
(EPS)

S6 Fig Correlation profile of the control gene-modules in the matched datasets. Correlation of the full-module scores of the control gene-modules computed from ref-1/ref-2/ref-3 reference dataset to the respective full-module/module-subset scores computed from the matched datasets. The panel A contains the heat map of the correlation profiles from the ref-1 comparison where the reference dataset was u133p2.3ivt.ff, and panel B and C contains similar plots from ref-2 and ref-3 comparisons. The reference datasets in ref-2 and ref-3 comparisons were hgu219.ovation.ff and hgu219.ovation.ffpe respectively. An arbitrary cutoff of correlation coefficient equal to 0.8 was used to distinguish a gene-module as concordant/reproducible or discordant/non-reproducible. The blue, red, and green colored boxplots beneath the heat map represent the distribution of module-score correlations before filtering, after PVAC_aab filtering and after PVAC_ag filtering respectively.
(EPS)

S7 Fig Influence of the module gene expression levels on the module-score correlation between matched datasets. Module-score correlation profiles of module-subsets derived from the full module based on genes/probesets PVAC filtering status or based on module genes mean expression distribution within each matched dataset. The plots represent expression profiles computed using ref-1 comparisons in non-filtered datasets; i.e., full module-scores from reference u133p2.3ivt.ff dataset is correlated with the matched dataset specific module-subset derived from the full module as mentioned above. The panels A, B, C, and D represent module-score correlation profiles from the matched datasets u133p2.3ivt.ffpe, u21.ovation.ff, u219.ovation.ffpe, and u219.sensation.ffpe respectively. A systematic higher correlation is observed in module-subsets with PVAC selected genes compared to module-subsets with PVAC non-selected genes.
(EPS)

S8 Fig Normalized expression distribution of PVAC selected and non-selected probesets. Blue color represents the PVAC non-selected probesets, and brown color represents PVAC selected probesets.
(EPS)

S9 Fig Influence of the gene-module coherence on the module-score correlation between matched datasets. Gene-module coherence was computed as the average pair-wise correlation of module genes per matched dataset. The module coherence value is plotted against module-score correlations between the reference and matched datasets in ref-1/ref-2/ref-3 comparisons. Only full-modules were considered in this analysis. The blue line represents a loess fit to the data points.
(EPS)

S10 Fig Influence of the module genes expression level on the module-score correlation between matched datasets. Gene-module average expression was computed as the grand average of average gene expression of module-genes per matched dataset. The average module expression value is plotted against module-score correlations between the reference and matched datasets in ref-1/ref-2/ref-3 comparisons. Only full-modules were considered in this analysis. The blue line represents a loess fit to the data points.
(EPS)

S11 Fig Influence of the module-score algorithm on the direction of the association between module coherence and the module-score correlation between matched datasets. Selected modules from external-module-set1 were used. Module-scores were computed from these modules as a weighted average or simple average, and figures similar to S9 Fig was generated to visualize the influence. Gene-module coherence was computed as the average pair-wise correlation of module genes per matched dataset. The module coherence value is plotted against module-score correlations between the reference and matched datasets in ref-1/ref-2/ref-3 comparisons. In panel A, module-score correlations were computed using module-scores calculated by simple-average, and in panel B module-score correlations were computed using module-scores calculated by weighted-average. Only full-modules were considered in this analysis. The blue line represents a loess fit to the data points.
(EPS)

S12 Fig Influence of the module-score algorithm on the direction of the association between module genes expression level and the module-score correlation between matched datasets. Selected modules from external-module-set1 were used. Module-scores were computed from these modules as a weighted average or simple average, and figures similar to S10 Fig was generated to visualize the influence. Gene-module average expression was computed as the grand average of average gene expression of module-genes per matched dataset. The average module expression value is plotted against module-score correlations between the reference and matched datasets in ref-1/ref-2/ref-3 comparisons. In panel A, module-score correlations were computed using module-scores calculated by simple-average, and in panel B module-score correlations were computed using module-scores calculated by weighted-average. Only full-modules were considered in this analysis. The blue line represents a loess fit to the data points.
(EPS)

S13 Fig Shared genes between different versions of the de-novo ER/HER2 gene-modules. A heat map of the percentage of shared genes between the different versions of the de-novo ER/HER2 gene-modules. Panel A represents de-novo modules derived from the non-filtered dataset, and panel B and C represents de-novo modules derived from PVAC_AAB filtered, and PVAC_AG filtered datasets respectively. The value of 'n' given in each cell of the heat map represents the number of shared genes between the respective gene-modules.
(EPS)

S14 Fig Distribution of percent-present values. Distribution of Percent-Present values A) in the matched datasets and B) in the external FFPE and frozen datasets used in this study.
(TIF)

S15 Fig Correlation profile of the external-module-set2 gene-modules in the matched datasets (linear scale). Module-score were computed from linear scale expression data. Correlation of the full-module scores of the external-module-set2 gene-modules (geneSigDB) computed from ref-1/ref-2/ref-3 reference dataset to the respective full-module/module-subset scores computed from the matched datasets. The panel A contains the heat map of the correlation profiles from the ref-1 comparison where the reference dataset was u133p2.3ivt.ff, and panel B and C contains similar plots from ref-2 and ref-3 comparisons. The reference datasets in ref-2 and ref-3 comparisons were hgu219.ovation.ff and hgu219.ovation.ffpe respectively. An arbitrary cutoff of correlation coefficient equal to 0.8 was used to distinguish a gene-module as concordant/reproducible or discordant/non-reproducible. The blue, red, and green colored boxplots beneath the heat map represent the distribution of module-score correlations before filtering, after PVAC_aab filtering and after PVAC_ag filtering respectively.
(EPS)

S1 Table Experiment design. (XLS)

S2 Table Sample demography. (XLS)

S3 Table PVAC_AAB selected probesets from each matched dataset. (XLS)

S4 Table PVAC_AG selected probesets from each matched dataset. (XLS)

S5 Table References of the external gene-modules considered. (XLS)

S6 Table External-module-set1 gene-module score correlation profile. (XLS)

S7 Table External-module-set2 gene-module score correlation profile. (XLS)

S8 Table Control gene-module score correlation profile. (XLS)

S1 File Supplementary methods. (DOC)
