# Supplementary figures and images for: Feasibility of developing reliable gene expression modules from FFPE derived RNA profiled on Affymetrix arrays
Source: PLoS One. 2018 Aug 31;13(8):e0203346. doi: 10.1371/journal.pone.0203346 (PMC6118369; doi:10.1371/journal.pone.0203346)

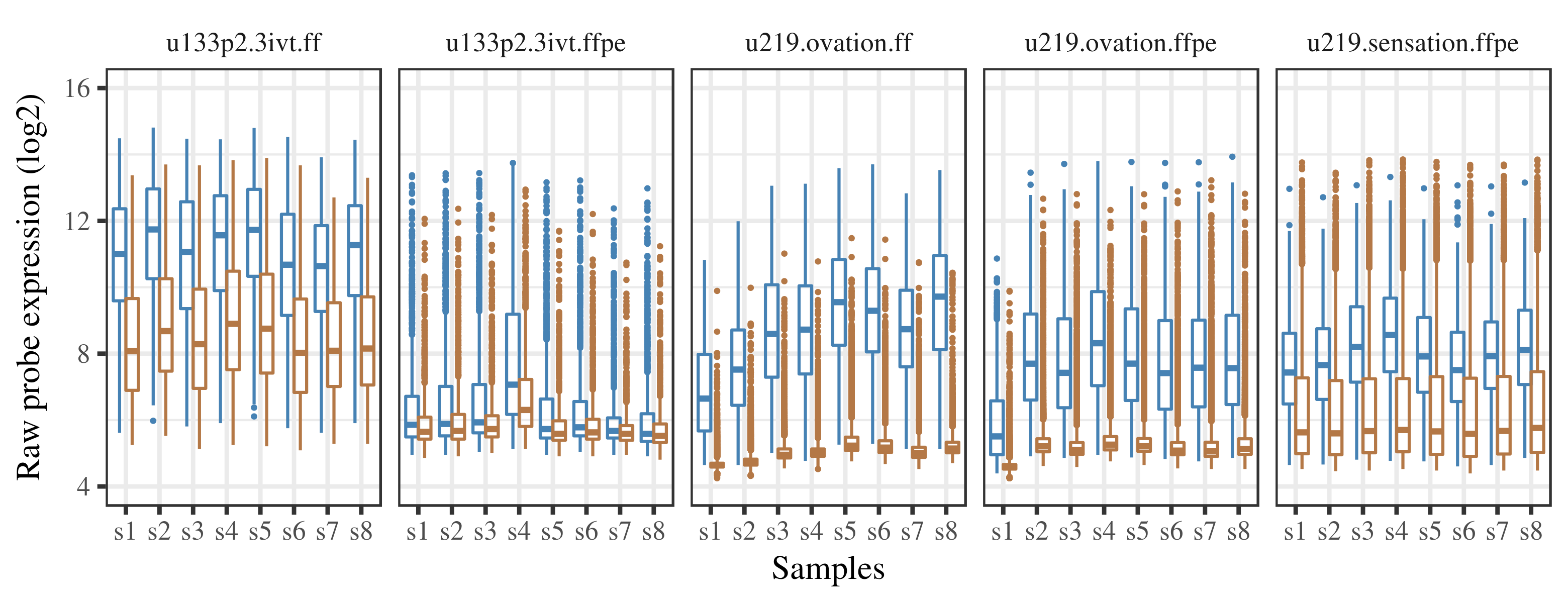

Supplement: S3 Fig — Blue color represents the raw PM probe expression of the 100 positive genomic control genes, and brown color represents the raw background expression. The background expression of the HG-U133plus2 array constitutes the MM probe expression of the 100 genomic positive control genes, and that of the HG-U219 array constitutes the expression of the 23 anti-genomic probesets. (TIFF) [file pone.0203346.s003.tiff]

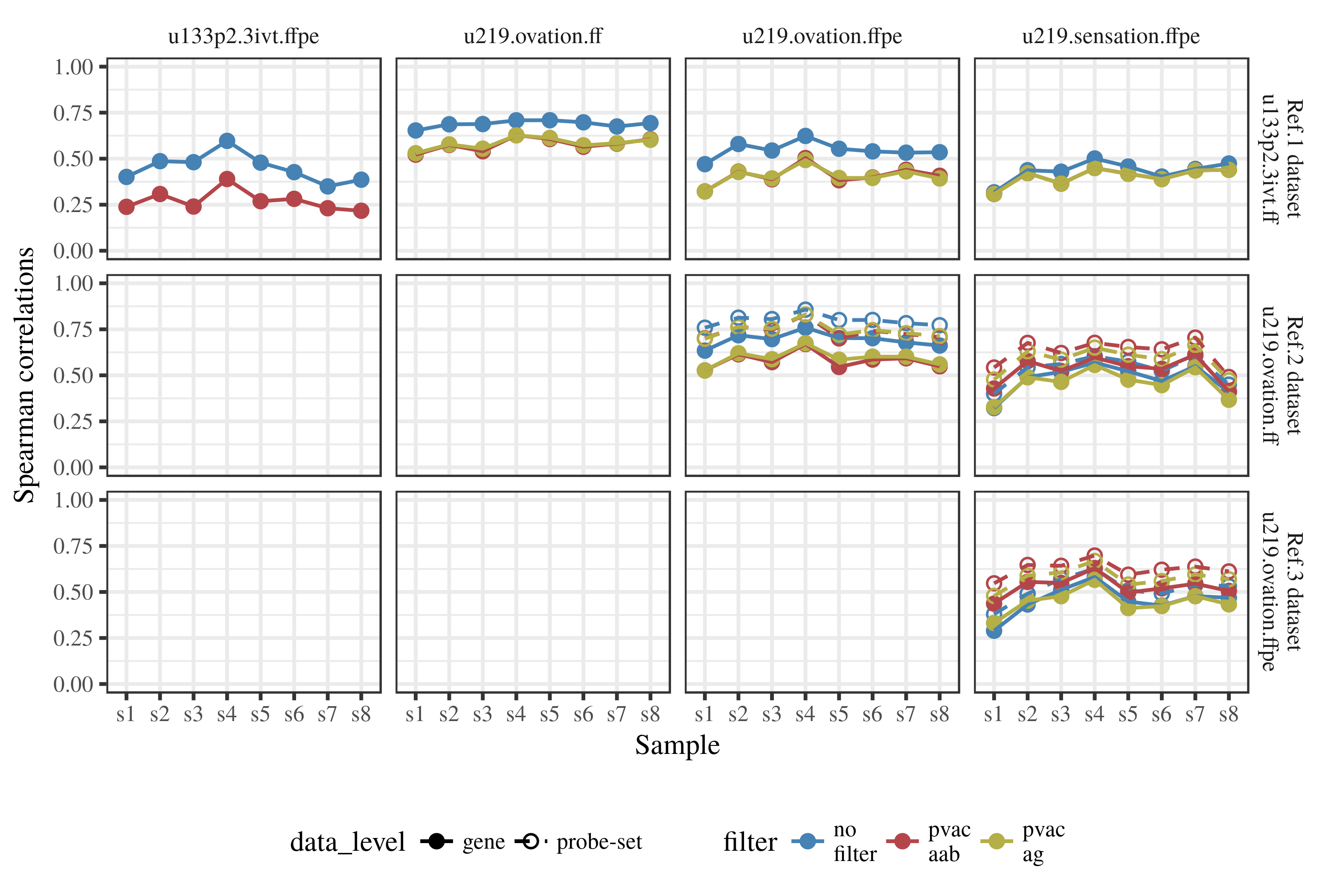

Supplement: S4 Fig — Blue, red and green color points and lines represent the distribution of sample correlations before filtering, after PVAC_aab filtering and after PVAC_ag filtering respectively. The open circle represents the distribution of sample correlation at the probeset level. The correlation coefficient is computed using common genes/probesets between reference datasets and matched datasets. (TIFF) [file pone.0203346.s004.tiff]

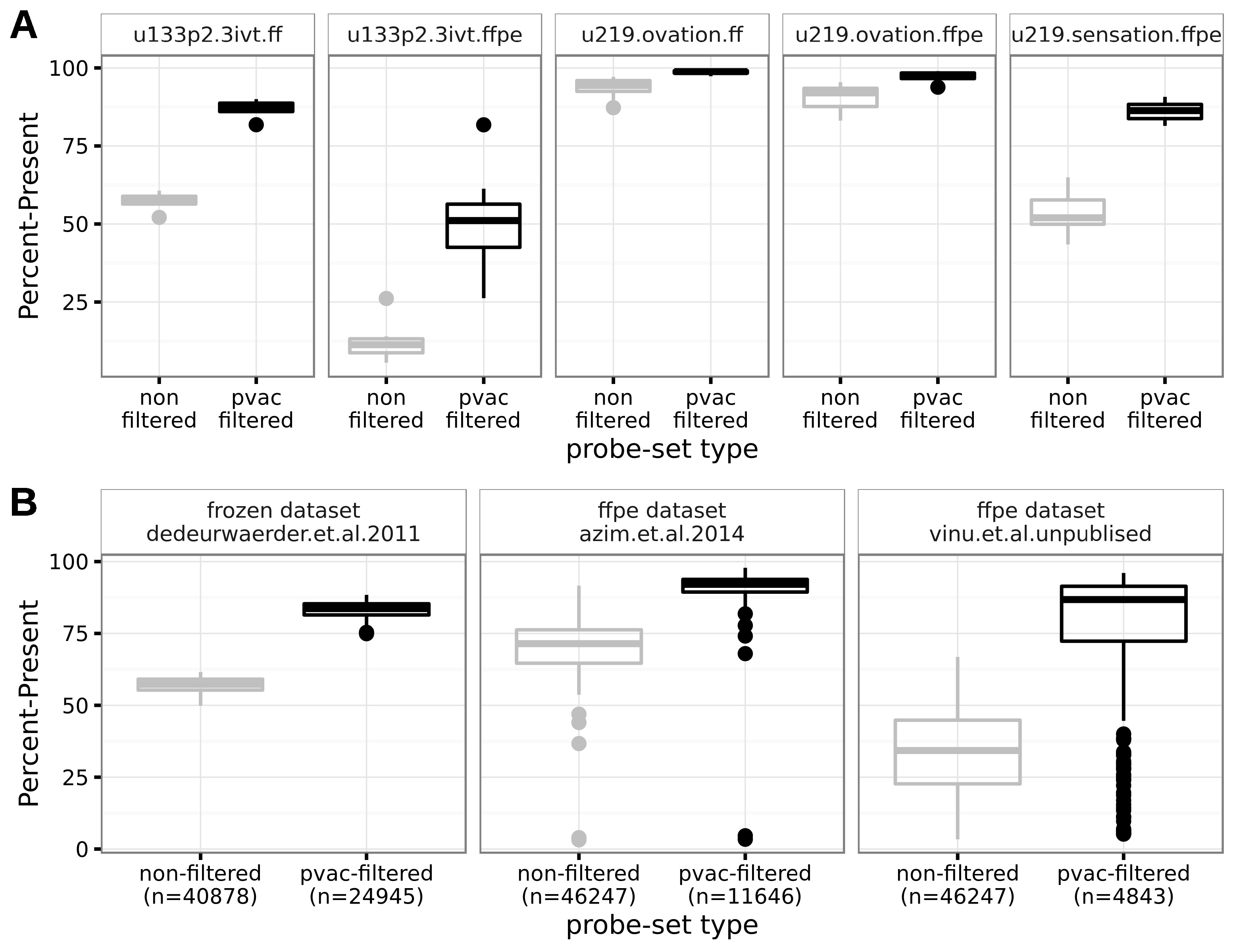

Supplement: S14 Fig — Distribution of Percent-Present values A) in the matched datasets and B) in the external FFPE and frozen datasets used in this study. (TIF) [file pone.0203346.s014.tif]
