# Supplementary material for: Feasibility of developing reliable gene expression modules from FFPE derived RNA profiled on Affymetrix arrays
Source: PLoS One. 2018 Aug 31;13(8):e0203346. doi: 10.1371/journal.pone.0203346 (PMC6118369; doi:10.1371/journal.pone.0203346)
Supplement: S1 File — (DOC) [file pone.0203346.s024.doc]

S1 File: Supplementary Methods

1. **Detection calling**
2. **Probe set filtering**
3. **External dataset processing**

# 1. Detection calling from the PM only HG-U219 arrays

In line with Affymetrix's high density whole transcript (WT) Exon 1.0 ST and Gene 1.0/2.0 ST arrays, the PM only HG-U219 array uses 23 GC-bin based anti-genomic (AG) probe sets (each AG probe set contains probes with same GC content) as a surrogate for measuring background and non-specific hybridization, instead of the paired MM probes [1,2]. A direct impact of this change is that the MM probe-based detection calling algorithm from MAS 5.0 package [3] can't be used with these arrays. Although Affymetrix introduced a new detection calling algorithm (DABG: Detection Above BackGround) for the exon level probe sets [2,4,5], it is not recommended to use directly on the gene level probe sets as the assumptions made in the algorithm could be violated [6]. Further, the probe design strategies were different for the gene and exon arrays [7,8]. Hence an alternate means to generate detection calls from the HG-U219 array was required.

Different algorithms for generating detection calls were considered which differ mainly in the selection of null/background distribution. The algorithms were listed below.

1. Use two-sample Wilcoxon rank sum test [9] to test whether the intensity distribution of the probes in a genomic probe set is shifted towards the right of the intensity distribution of the probes of relevant anti-genomic probe sets (null distribution). Different strategies for selecting appropriate null distribution were listed below:
   1. Null distribution as the probe intensity of the AG probe set with GC content equal to the **median GC content** of probes in the genomic probe set being considered **(MedGC)**.
   2. Null distribution as the probe intensity of the AG probe set with GC content equal to the **highest GC content** of the probes in the genomic probe set being considered **(MaxGC)**.
   3. Null distribution as the probe intensity of the AG probe set with **pre-specified GC content (FixGC)** which defaults to the median GC content (i.e., 12 and 13) of the entire PM probes in the array
   4. Null distribution as the probe intensity of all AG probe set with GC content equal to the **unique GC content** of probes in the genomic probe set being considered **(RelGC)**.
   5. Null distribution as the probe intensity of **all 23 AG probe sets** present in the array **(AllGC)**.
2. Use MAS5 algorithm [3] and replace the MM-probe intensity with the median probe intensity of the AG probe set with GC content equal to the corresponding PM-probe's GC content **(MAS5GC)**.

Each of the detection calling algorithms will give a p-value associated with each probe set. However, the default 5% significance level of detection calling algorithm from MAS 5.0 package [3] may not be appropriate for the methods mentioned above. Among the three matched HG-U219 datasets (see the main article), an optimal significance level is determined by using the sensitivity-specificity analysis, considering the MAS5 detection calls from the u133p2.3ivt array with frozen samples (u133p2.3ivt.ff) as the 100% truth. Instead of using an ad-hoc approach to determine optimal significance level, only standard significance levels were considered in this analysis; i.e 5% (0.05), 1% (0.01) and 0.1% (0.001).


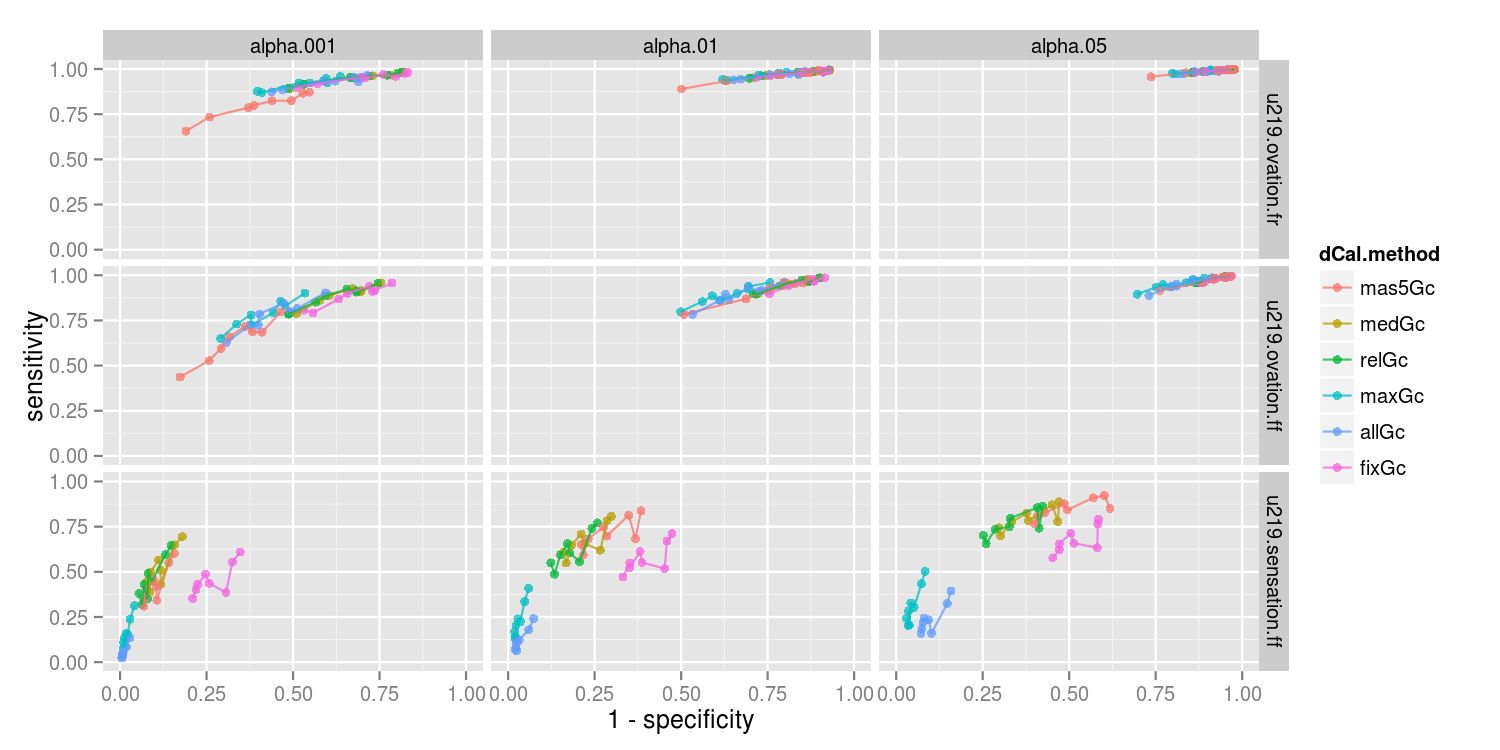


**Fig A: Sensitivity specificity analysis of detection-calling algorithms.** Sensitivity-specificity analysis to find the best detection calling algorithm and an optimal significance level for HG-U219 arrays. To compute the sensitivity-specificity value for each sample, present-absent calls of each sample (generated using a single detection calling algorithm at a specific significance level) from each HG-U219 dataset, was compared to MAS present-absent calls generated at 5% significance level from the matching reference sample (100% truth). Each point in the figure represents the sensitivity-specificity value of a single sample computed using the above procedure. The eight sensitivity specificity values obtained from the eight matched samples of a single HG-U219 dataset using a single HG-U219 detection calling algorithm were joined by a solid line to simplify interpretation. The vertical panel represents the significance level (alpha level) used to generate present-absent calls from each matched HG-U219 datasets using the different detection calling algorithms (color coded), and horizontal panel represents each matched HG-U219 datasets. u219.ovation.fr represents frozen HG-U219 dataset with ovation kit,u219.ovation.ff represents FFPE HG-U219 dataset with ovation kit and u219.sensation.ff represents FFPE HG-U219 dataset with sensation kit.

The output of the sensitivity-specificity analysis was summarized in Fig A. The unrealistically low background in HG-U219 datasets with ovation kit (see Fig 1 of main paper ) resulted in high sensitivity and low specificity irrespective of the different detection calling algorithms and significance levels considered. However HG-U219 dataset with sensation kit was sensitive to the different detection call algorithms and significance levels considered. Specifically, with MaxGC and AllGC, the extreme detection call algorithms among the others, very low sensitivity was observed at all significance levels. Among the remaining algorithms MAS5GC, MedGC and RelGC showed similar performance, and the optimal sensitivity and specificity were observed at significance level 0.01. However, due to simplicity in implementation and interpretation, the MedGC algorithm at alpha level 0.01 was used in this analysis and is recommended for the HG-U219 array with sensation kit. Further detection calling from HG-U219 arrays with ovation kit may not be reliable due to the unrealistically low background observed in these datasets.

# 2. P**robe set** filtering

In expression datasets derived from FFPE samples, a high range of percent-present (pp) values has been reported [10–15]. This high range of pp value may pose a problem in analyzing FFPE expression profiles generated from Affymetrix arrays, as Affymetrix recommends similar pp values between samples in a dataset [16]. Further, it could also be due to the variation in the degree of RNA degradation present in the FFPE samples. So we cannot consider all the probe sets from FFPE expression profiles as reliable and strict filtering before analyzing this kind of dataset is unavoidable. However, we cannot use a majority of the probe set filtering strategies, as they all developed considering the comparable pp values of expression profiles from the frozen samples in mind. For instance, probe set filtering methods which solely depends on present-absent calls, such as the all-absent [17] and fraction-present based [18] filtering, cannot be applied to expression datasets derived from FFPE samples with non-comparable present-absent distribution. Further, the variance based filtering [17] may not be appropriate for the FFPE expression profiles, as by construction variance filtering always selects a fixed percentage of probe sets (say 50%) from every dataset as reliable. However, the per probe-set probe agreement based probeset filtering from Lu et al. (PVAC) [19] seems to be well suited for expression profiles derived from degraded RNA. But this method depends on the all-absent probe set for generating dataset depended cutoff to select reliable probe sets (PVAC_AAB). Using the all-absent probesets for defining a cutoff would be fine, but it is unreliable for probe set filtering from the FFPE expression profiles. For instance in a dataset with high range of pp values, say between 20% and 65%, the all-absent will be sensitive to the absent call from high pp samples (i.e., 65%) and identify at most 35% absent probe sets from this array as unreliable for the entire dataset; however this all-absent method does not account for the 45% (high pp - low pp; 65-20=45% ), absent probe sets in sample with 20%pp values. In this analysis, we used a custom R implementation of PVAC method, as the algorithm provided in Lu et al. [19] doesn't support PM only arrays, such as the HG-U219 arrays used in this study. The PVAC algorithm was applied to background corrected and normalized datasets, as recommended by the Lu et al. [19]. We also investigated the possibility of using the 23 anti-genomic probesets present in the HG-U219 array as negative probesets for PVAC filtering (PVAC_AG).

# 3. External dataset processing

For the FFPE HG-U219 (GSE53031) [20] and frozen HG-U133plus2 (GSE20713) [21] external datasets, detection calls were generated from raw CEL files using MedGC (see detection calling section) and MAS5 [3,22] detection calling algorithms respectively. We used the significance level of 0.01 for MedGC and 0.05 for MAS5 for generating present-absent calls. A custom R implementation of the PVAC method detailed in Lu et al. [19] was used to select reliable probe sets from the RMA background-corrected and quantile-normalized datasets. Normalized expression summaries were extracted from series matrix available under the respective GEO accession numbers.

# References

1. Affymetrix, Inc. Human Genome U219 Array Plate [Internet]. Santa Clara (CA): Affymetrix; c2009-2010 [cited 2016 Dec 14]. Part No.: 702857 Rev. 3. Available from: http://media.affymetrix.com/support/downloads/package_inserts/hgu219_ap_insert.pdf

2. Affymetrix Inc. Exon Array Background Correction [Internet]. Santa Clara (CA): Affymetrix; 2005 Sep [cited 2016 Sep 2]. 5 p. Available from: http://www.affymetrix.com/support/technical/whitepapers/exon_background_correction_whitepaper.pdf

3. Liu W–., Mei R, Di X, Ryder TB, Hubbell E, Dee S, et al. Analysis of high density expression microarrays with signed-rank call algorithms. Bioinformatics. 2002 Dec 1;18(12):1593–9.

4. Kapur K, Xing Y, Ouyang Z, Wong WH. Exon arrays provide accurate assessments of gene expression. Genome Biol. 2007;8:R82.

5. Affymetrix Inc. Exon Array Analysis: What is DABG, PLIER? [Internet]. Santa Clara (CA): Affymetrix [cited 2016 Sep 4]. Available from: http://www.affymetrix.com/support/help/faqs/exon_array_analysis/faq_8.jsp

6. Affymetrix Inc. Identifying and Validating Alternative Splicing Events [Internet]. Santa Clara (CA): Affymetrix; c2006 [cited 2016 Sep 4] 16 p. Part No.: 702422 Rev. 1. Available from: http://www.affymetrix.com/support/technical/technotes/id_altsplicingevents_technote.pdf

7. Affymetrix Inc. Array Design for the GeneChip® Human Genome U133 Set [Internet]. Santa Clara (CA): Affymetrix; c2001-2007 [cited 2016 Sep 4] 12 p. Part No.: 701133 Rev. 2. Available from: http://media.affymetrix.com/support/technical/technotes/hgu133_design_technote.pdf

8. Affymetrix Inc. GeneChip® Exon Array Design [Internet]. Santa Clara (CA): Affymetrix; c2005 [cited 2016 Sep 4] 10 p. Part No.: 702026 Rev. 1. Available from: http://media.affymetrix.com/support/technical/technotes/exon_array_design_technote.pdf

9. Hollander M, Wolfe DA. Nonparametric Statistical Methods. 1^St^ ed. New York: Wiley; 1973. 528 p.

10. Budczies J, Weichert W, Noske A, Müller BM, Weller C, Wittenberger T, et al. Genome-wide gene expression profiling of formalin-fixed paraffin-embedded breast cancer core biopsies using microarrays. J Histochem Cytochem. 2011;59(2):146–57.

11. Lassmann S, Kreutz C, Schoepflin A, Hopt U, Timmer J, Werner M. A novel approach for reliable microarray analysis of microdissected tumor cells from formalin-fixed and paraffin-embedded colorectal cancer resection specimens. J Mol Med. 2009 Feb;87(2):211–24.

12. Linton K, Hey Y, Dibben S, Miller C, Freemont A, Radford J, et al. Methods comparison for high-resolution transcriptional analysis of archival material on Affymetrix Plus 2.0 and Exon 1.0 microarrays. BioTechniques. 2009 Jul;47(1):587–96.

13. Roberts L, Bowers J, Sensinger K, Lisowski A, Getts R, Anderson MG. Identification of methods for use of formalin-fixed, paraffin-embedded tissue samples in RNA expression profiling. Genomics. 2009 Nov;94(5):341–8.

14. Thomas M, Poignée-Heger M, Weisser M, Wessner S, Belousov A. An optimized workflow for improved gene expression profiling for formalin-fixed, paraffin-embedded tumor samples. J Clin Bioinforma. 2013 May 3;3:10.

15. Williams PM, Li R, Johnson NA, Wright G, Heath J-D, Gascoyne RD. A Novel Method of Amplification of FFPET-Derived RNA Enables Accurate Disease Classification with Microarrays. J Mol Diagn JMD. 2010 Sep;12(5):680–6.

16. Affymetrix Inc. GeneChip® Expression Analysis - Data Analysis Fundamentals [Internet]. Santa Clara (CA): Affymetrix; c2002-2004 [cited 2016 Sep 2]. 108 p. Part No.: 701190 Rev. 4. Available from: http://media.affymetrix.com/support/downloads/manuals/data_analysis_fundamentals_manual.pdf

17. Hackstadt AJ, Hess AM. Filtering for increased power for microarray data analysis. BMC Bioinformatics. 2009;10:11.

18. McClintick JN, Edenberg HJ. Effects of filtering by Present call on analysis of microarray experiments. BMC Bioinformatics. 2006;7:49.

19. Lu J, Kerns RT, Peddada SD, Bushel PR. Principal component analysis-based filtering improves detection for Affymetrix gene expression arrays. Nucleic Acids Res. 2011 Jul;39(13):e86.

20. Azim HA, Brohée S, Peccatori FA, Desmedt C, Loi S, Lambrechts D, et al. Biology of breast cancer during pregnancy using genomic profiling. Endocr Relat Cancer. 2014 Aug 1;21(4):545–54.

21. Dedeurwaerder S, Desmedt C, Calonne E, Singhal SK, Haibe-Kains B, Defrance M, et al. DNA methylation profiling reveals a predominant immune component in breast cancers. EMBO Mol Med. 2011 Dec;3(12):726–41.

22. Gautier L, Cope L, Bolstad BM, Irizarry RA. affy—analysis of Affymetrix GeneChip data at the probe level. Bioinformatics. 2004 Feb 12;20(3):307–15.
